# A Mixture of Extracts of *Kochia scoparia* and *Rosa multiflora* with PPAR α/γ Dual Agonistic Effects Prevents Photoaging in Hairless Mice

**DOI:** 10.3390/ijms17111919

**Published:** 2016-11-16

**Authors:** Hyerin Jeon, Dong Hye Kim, Youn-Hwa Nho, Ji-Eun Park, Su-Nam Kim, Eung Ho Choi

**Affiliations:** 1 Department of Dermatology, Yonsei University Wonju College of Medicine, Wonju 26426, Korea; moonlady84@naver.com (H.J.); donghye05@hanmail.net (D.H.K.); 2 Skin Research Team, Cosmax R&I Center, Seongnam 13486, Korea; yhno@cosmax.com (Y.-H.N.); jepark@cosmax.com (J.-E.P.); 3 Natural Skinomics Team, KIST Gangneung Institute of Natural Products, Gangneung 25451, Korea; snkim@kist.re.kr

**Keywords:** PPAR α/γ, photoaging, ultraviolet radiation, skin barrier, procollagen 1

## Abstract

Activation of peroxisome proliferator-activated receptors (PPAR) α/γ is known to inhibit the increases in matrix metalloproteinase (MMP) and reactive oxygen species (ROS) induced by ultraviolet light (UV). Extracts of natural herbs, such as *Kochia scoparia* and *Rosa multiflora*, have a PPAR α/γ dual agonistic effect. Therefore, we investigated whether and how they have an antiaging effect on photoaging skin. Eighteen-week-old hairless mice were irradiated with UVA 14 J/cm^2^ and UVB 40 mJ/cm^2^ three times a week for 8 weeks. A mixture of extracts of *Kochia scoparia* and *Rosa multiflora* (KR) was topically applied on the dorsal skin of photoaging mice twice a day for 8 weeks. Tesaglitazar, a known PPAR α/γ agonist, and vehicle (propylene glycol:ethanol = 7:3, *v*/*v*) were applied as positive and negative controls, respectively. Dermal effects (including dermal thickness, collagen density, dermal expression of procollagen 1 and collagenase 13) and epidermal effects (including skin barrier function, epidermal proliferation, epidermal differentiation, and epidermal cytokines) were measured and compared. In photoaging murine skin, KR resulted in a significant recovery of dermal thickness as well as dermal fibroblasts, although it did not change dermal collagen density. KR increased the expression of dermal transforming growth factor (TGF)-β. The dermal effects of KR were explained by an increase in procollagen 1 expression, induced by TGF-β, and a decrease in MMP-13 expression. KR did not affect basal transepidermal water loss (TEWL) or stratum corneum (SC) integrity, but did decrease SC hydration. It also did not affect epidermal proliferation or epidermal differentiation. KR decreased the expression of epidermal interleukin (IL)-1α. Collectively, KR showed possible utility as a therapeutic agent for photoaging skin, with few epidermal side effects such as epidermal hyperplasia or poor differentiation.

## 1. Introduction

Skin aging is divided into intrinsic aging that occurs unavoidably as a result of chronological aging, and extrinsic aging that occurs as a result of exposure to factors in the external environment, such as ultraviolet light [1,2]. Intrinsic aging is usually observed in unexposed skin, while extrinsic aging, including photoaging, represents the cumulative effects of intrinsic aging and chronic light exposure on sun-exposed skin, such as the face, the dorsum of the hand, and the posterior neck [3].

There are many agents used to treat aging skin and wrinkles. Among them, retinoid, which is excellent for improving wrinkles, is the only drug approved by US Food and Drug Administration (FDA) for the treatment of skin aging. However, it has notable side effects, including erythema, a burning sensation, pruritus, and scaling. Since retinoid can aggravate xerosis and epidermolysis when it is used on aging skin, particular attention is required [4]. Botulinum toxin and hyaluronate filler can cause pain and damage to skin, and are more expensive than retinoid. Therefore, safer and more rational antiaging products are continuously needed [5].

Nuclear hormone receptors (NHR) are types of transcription factors activated by ligands. Unlike extracellular receptors that react to peptide ligands, NHRs move to the target gene in the nucleus after binding to lipophilic hormones or ligands that have entered the cell and then regulate transcription or the gene’s expression [6]. It has been reported that 48 NHR family members are encoded in humans, according to genomic sequences. They are generally classified according to the types of hormones or ligands binding to the receptors [7]. Most of the research on the NHR family has primarily focused on the treatment and prevention of metabolic syndromes, including diabetes, obesity, hypertension, and hyperlipidemia. On the other hand, as it has been continuously elucidated that NHR plays an important role in the pathophysiology related to aging, active research is ongoing to develop activating materials of NHRs and elucidate their mechanisms of action.

The NHRs affecting skin function and metabolism include the thyroid hormone receptor-like (RAR, PPAR, RPR, LXR, FXR, and VDR), retinoid X receptor-like (RXR), and estrogen-like (EF) receptors [8,9]. Among these, peroxisome proliferator-activated receptor (PPAR) agonists and liver X receptor (LXR) agonists have been studied as means of alleviating skin inflammation and improving barrier function, based on the anti-inflammatory effect of NHRs [10,11,12]. All isotypes of PPARs (PPAR α, PPAR β/δ, PPAR γ) have been reported to reduce inflammation, accelerate epidermal differentiation, inhibit epidermal proliferation, and enhance skin barrier function [12]. In an animal model of atopic dermatitis (AD), a PPAR α agonist was shown to alleviate skin inflammation, suppress the increase in epidermal thickness, and reduce inflammatory cells in the dermis, thereby improving AD [13]. The PPAR α agonist also prevented the skin barrier impairment, the decrease in skin thickness and epidermal differentiation markers, and the weakness of the skin barrier caused by topical glucocorticoids [14]. PPAR γ is expressed in dermal fibroblasts and PPAR γ agonists, such as troglitazone, suppress the biosynthesis of matrix materials, such as collagen I or fibronectin, by inhibiting transforming growth factor (TGF)-β, a major factor in fibrosis [15]. Rosiglitazone (PPAR γ agonist) increases the expression of matrix metalloproteinase (MMP)-3 and MMP-13, while it decreases the expression of tissue inhibitor of metalloprotease (TIMP)-1 [16]. Furthermore, it was reported that 5,7-dimethoxyflavone, an activator of PPAR α/γ, not only inhibits the increase in MMP-1, MMP-2, MMP-9, and ROS induced by UV light in fibroblasts, but also suppresses inflammatory cytokines, such as interleukin (IL)-6 and IL-8 [17].

*Kochia scoparia* is a large annual herb in the family Amaranthaceae and is native to Eurasia. It grows commonly in China, Japan, and Korea, and its mature fruit has been used throughout the area in traditional medicine to treat diseases including skin problems and inflammatory and allergic diseases [18]. *Rosa multiflora* is a species of rose native to Eastern Asia, such as China, Japan, and Korea. It has been traditionally used in China as a dietary supplement and herbal remedy to treat diseases such as osteoarthritis, rheumatoid arthritis, inflammation, and chronic pain [19]. While searching for natural herbs with antiaging effects, of eight kinds of natural herbs, *Kochia scoparia* and *Rosa multiflora* were selected via in vitro screening tests. Then, through in vitro tests, we confirmed that *Kochia scoparia* and *Rosa multiflora* had PPAR α/γ dual agonistic effects. Therefore, we chose these two plants’ extracts for antiaging studies.

In this study, we used their mixture, instead of an individual plant extract, because we expected there to be a synergy between the two plants. We investigated whether and how a mixture of extracts of *Kochia scoparia* and *Rosa multiflora*, which was confirmed to have a PPAR α/γ dual agonistic effect in an in vitro study, results in an antiaging effect on photoaging murine skin.

## 2. Results

### 2.1. Dual Activation of PPAR α/γ Transcriptional Activity by Kochia scoparia (Ks) and Rosa multiflora (Rm)

**Figure 1:**
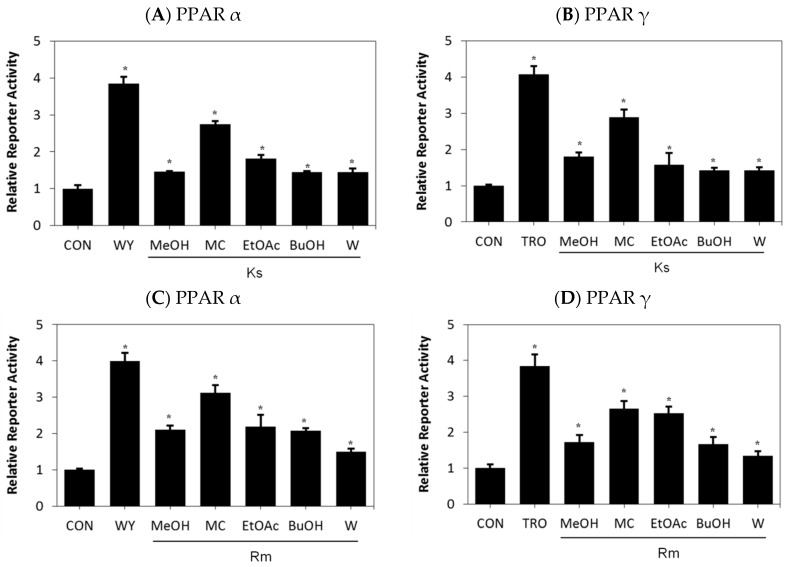
Peroxisome proliferator-activated receptor (PPAR) transcriptional activity after treatment with extracts of *Kochia scoparia* (Ks) or *Rosa multiflora* (Rm) in CV-1 cells, a line of monkey kidney cells. CV-1 cells were transfected with human PPAR α (**A**,**C**) or PPAR γ (**B**,**D**) expression vectors, the peroxisome proliferator response element (PPRE)–luciferase reporter construct, and the pRL-SV40 vector. After transfection, cells were treated with various extracts and fractions of Ks (**A**,**B**) or Rm (**C**,**D**) for 24 h and the relative transcriptional activities were analyzed. WY: WY14643 acting primarily as an activator of PPAR α, TRO: Troglitazone acting as a ligand for both PPAR α and more strongly for PPAR γ, MeOH: Methanol extract, MC: Methylene chloride, EtOAc: Ethyl acetate, BuOH: Butanol, W: water. Each bar represents the mean ± SEM of duplicates. * *p* < 0.05 vs. control.

To explore the pharmacological properties of *Kochia scoparia* (Ks) and *Rosa multiflora* (Rm), we examined the effects of Ks and Rm on transcriptional activation of PPARs. Treatment with the methylene chloride (MC) fractions of Ks and Rm led to an increase in both PPAR α- and PPAR γ-reporter gene activities (Figure 1A–D); however, they minimally affected the transcriptional activation of PPAR δ and did not have an effect on the RXR α (data not shown). These results strongly indicate the presence of a novel PPAR α/γ agonist in the extracts of Ks and Rm.

### 2.2. Kochia scoparia (Ks) and Rosa multiflora (Rm) Inhibit UVB-Induced MMP-1 Expression

**Figure 2:**
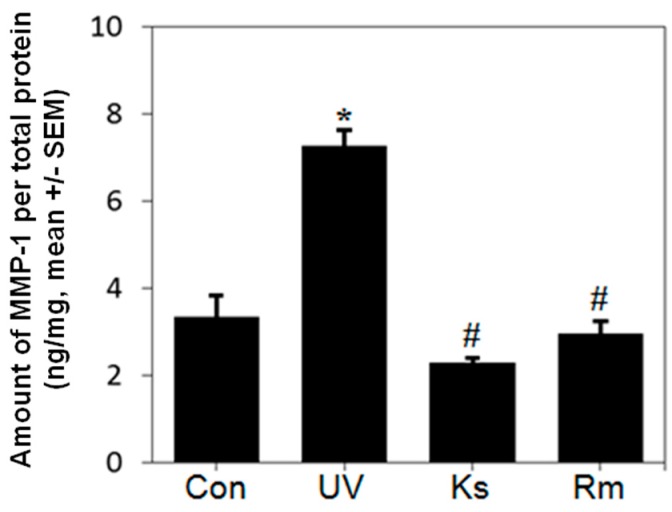
*Kochia scoparia* (Ks) and *Rosa multiflora* (Rm) inhibit UVB-induced MMP-1 expression. Normal human fibroblast (NHF) cells were treated with the methylene chloride (MC) fraction of Ks or Rm (10 µg/mL for each) for 1 h, exposed to UVB irradiation (20 mJ/cm^2^) and treated with 10 µg/mL of the MC fraction of Ks or Rm for 48 h to measure MMP-1 expression. Relative MMP-1 protein expression in Ks- or Rm-treated NHF cells was determined by ELISA. Ks and Rm significantly inhibited MMP-1 secretion in UVB-irradiated NHF cells. Con: sham light control, UV: UVB irradiation, Ks: *Kochia scoparia* treatment, Rm: *Rosa multiflora* treatment. Each bar represents the mean ± SEM of duplicates. * *p* < 0.05 vs. control; # *p* < 0.05 vs. UV.

To determine the effects of Ks and Rm on UVB-induced MMP-1 secretion, we quantified MMP-1 protein levels by ELISA using supernatants from Ks- or Rm-treated cell cultures. Ks and Rm significantly inhibited MMP-1 secretion in UVB-irradiated NHF cells (Figure 2). These findings suggest that the methanol chloride (MC) fractions of Ks and Rm block MMP-1 protein production.

### 2.3. Dermal Effect of a Mixture of Extracts of Kochia scoparia and Rosa multiflora (KR) in Photoaging Murine Skin

**Figure 3:**
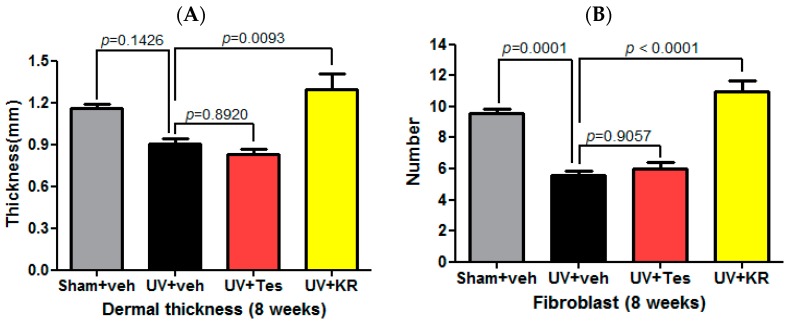
Effects of a mixture of extracts of *Kochia scoparia* and *Rosa multiflora* (KR) on the dermis in photoaging murine skin. Dermal effects were assessed by measuring dermal thickness (**A**) and counting the number of fibroblasts (**B**) in photoaging skin treated with topical KR, vehicle (veh), or tesaglitazar (Tes) for 8 weeks. Dermal thickness was defined as the distance from the subcutaneous fat to the dermal–epidermal junction. UV radiation decreased fibroblasts and dermal thickness. In photoaging skin, dermal thickness decreased, although not significantly, and dermal fibroblasts decreased significantly compared to vehicle. KR resulted in a significant recovery of dermal thickness and dermal fibroblasts in photoaging skin, whereas Tes was unable to cause these effects.

**Figure 4:**
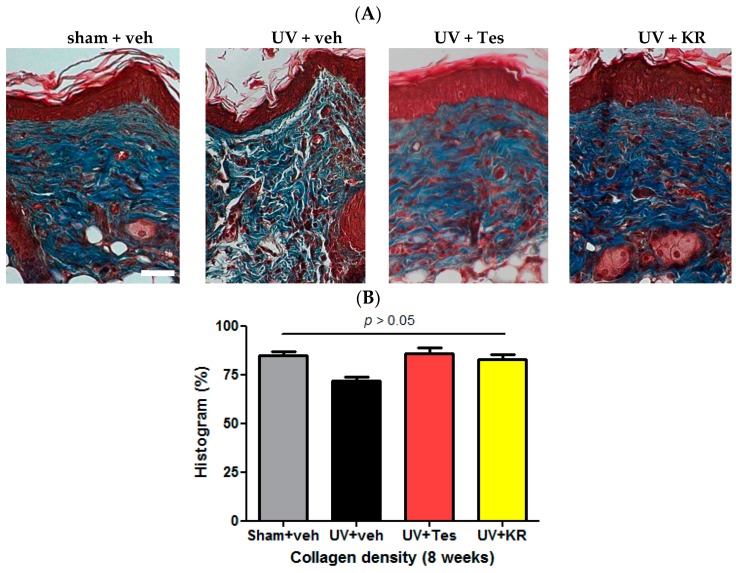
Effect of a mixture of extracts of *Kochia scoparia* and *Rosa multiflora* (KR) on dermal collagen density in photoaging murine skin. To evaluate collagen density in the dermis, Masson-trichrome staining was performed in photoaging skin treated with topical KR, vehicle (veh), or tesaglitazar (Tes) for 8 weeks (**A**); we used the histogram function of Photoshop^®^ software to evaluate collagen density (**B**). In photoaging murine skin, dermal collagen densities did not significantly change with either KR or Tes treatment; Scale bar = 100 µm.

To evaluate the dermal effects of KR in photoaging skin, we measured dermal thickness and counted the number of fibroblasts using hematoxylin and eosin (H & E) staining (Figure 3A,B), and determined dermal collagen density using Masson-trichrome staining. Tesaglitazar (Tes), a known PPAR α/γ dual agonist, was used as a positive control agent (Figure 4A). UV radiation decreased dermal thickness, although the decrease was not statistically significant, and significantly decreased dermal fibroblasts compared to sham light (Figure 3A,B). KR caused a significant recovery of dermal thickness as well as dermal fibroblasts in photoaging skin, whereas Tes did not have these effects (Figure 3A,B). However, UV radiation did not change dermal collagen densities. In photoaging skin, neither KR nor Tes affected dermal collagen density (Figure 4).

### 2.4. Dermal Effects of KR in Photoaging Skin Were Explained by an Increase in Procollagen 1 Expression, Induced by TGF-β, and a Decrease in MMP-13 Expression

**Figure 5:**
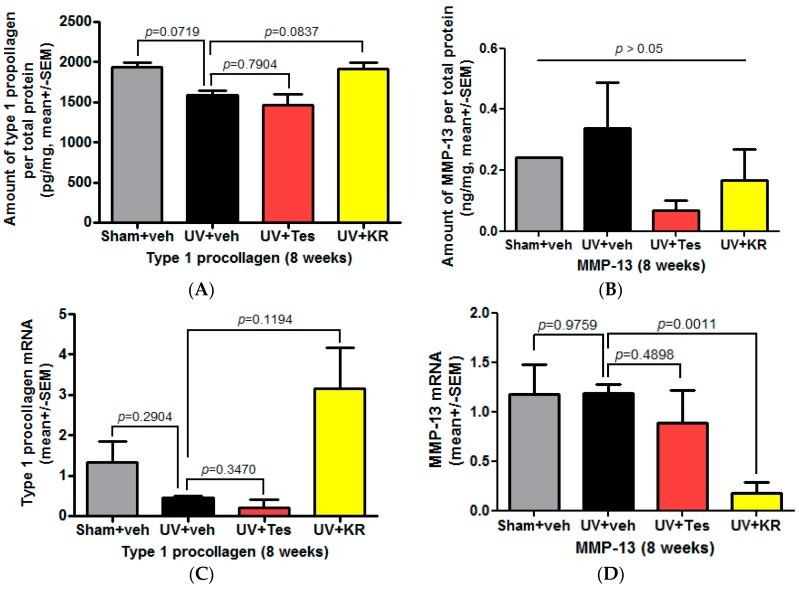
Effect of a mixture of extracts of *Kochia scoparia* and *Rosa multiflora* (KR) on dermal expression of procollagen and collagenase in photoaging murine skin. The expression of collagen and collagenase in photoaging skin, represented by procollagen type 1 and matrix metalloproteinase (MMP)-13, was determined by ELISA after topical application of KR, vehicle (veh), or tesaglitazar (Tes) for 8 weeks. In photoaging skin, the expression of type 1 procollagen protein (**A**) and mRNA (**C**) levels decreased; this decrease was not significant. KR increased these levels while Tes did not (**A**,**C**). On the other hand, expression of MMP-13 did not change (**B**,**D**). However, KR, but not Tes, significantly decreased the expression of MMP-13 mRNA (**D**).

**Figure 6:**
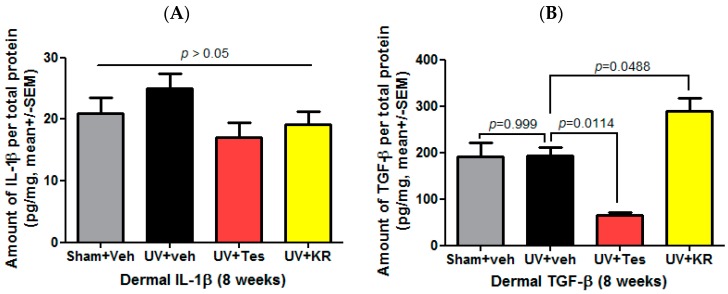
Effect of a mixture of extracts of *Kochia scoparia* and *Rosa multiflora* (KR) on cytokine expression in the dermis of photo-aging murine skin. Dermal expression levels of interleukin (IL)-1β and transforming growth factor (TGF)-β were determined by a cytokine assay using a Milliplex kit after topical application of KR, vehicle (veh), or tesaglitazar (Tes) for 8 weeks. In photoaging skin, dermal IL-1β (**A**) and TGF-β (**B**) did not change. KR significantly increased dermal TGF-β (**B**), but not dermal IL-1β (**A**).

To investigate whether KR protects the dermis in photoaging skin, we confirmed the expression of collagen and collagenase in the dermis, as evidenced by procollagen type 1 and matrix metalloproteinase-13 (MMP-13) expression, by ELISA. In photoaging skin, collagen expression decreased compared to skin exposed to sham light (Figure 5A). KR increased the protein and mRNA levels of type 1 procollagen relative to levels seen in vehicle-treated skin, although these increases were not significant (Figure 5A,C). On the other hand, the expression of MMP-13 did not change compared to skin exposed to sham light (Figure 5B,D). However, in photoaging skin, KR, but not Tes, significantly decreased the expression of MMP-13 mRNA (Figure 5D). These results demonstrated that KR increased collagen production, which was induced by TGF-β increase (Figure 6B), and decreased the expression of collagenase in photoaging murine skin. These findings indicate that KR restores the activity of dermal fibroblasts weakened by chronic UV radiation and, in turn, promotes collagen synthesis, whereas it inhibits MMP-mediated collagen degradation.

### 2.5. A Mixture of Extracts of Kochia scoparia and Rosa multiflora (KR) Increased the Expression of Dermal TGF-β in Photo-Aging Murine Skin

To measure cytokine expression in photoaging murine skin, we confirmed the expression of dermal IL-1β and dermal TGF-β. UV radiation did not change the expression of dermal TGF-β. In photoaging skin, KR significantly increased dermal TGF-β expression compared to vehicle, while Tes significantly decreased it (Figure 6B). In addition, KR and Tes did not affect the expression of dermal IL-1β in photoaging skin (Figure 6A).

### 2.6. A Mixture of Extracts of Kochia scoparia and Rosa multiflora (KR) Did Not Affect Basal Transepidermal Water Loss (TEWL) and Stratum Corneum (SC) Integrity, But Did Decrease SC Hydration in Photoaging Murine Skin

**Figure 7:**
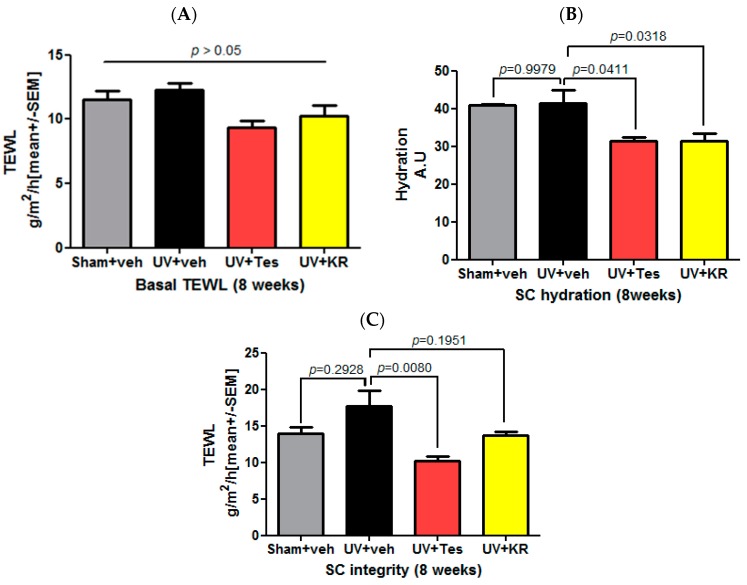
Effect of a mixture of extracts of *Kochia scoparia* and *Rosa multiflora* (KR) on the epidermal permeability barrier in photoaging skin. Basal transepidermal water loss (TEWL), stratum corneum (SC) hydration, and SC integrity were measured after UV radiation and topical application of KR, vehicle (veh), or tesaglitazar (Tes) for 8 weeks. An 8-week course of UV radiation on 18-week-old mice did not significantly affect basal TEWL, SC hydration, and SC integrity. In photoaging skin, neither KR nor Tes affected basal TEWL (**A**), but both decreased SC hydration significantly (**B**). To evaluate SC integrity, we measured TEWL after four sequential strippings with D-squame^®^. KR slightly increased SC integrity in photoaging skin, while Tes increased it significantly (**C**).

Basal TEWL, SC hydration, and SC integrity were measured in order to investigate the effects of KR on epidermal barrier function. An 8-week course of UV radiation did not change basal TEWL, SC hydration, and SC integrity (Figure 7). In photoaging skin, neither KR nor Tes affected basal TEWL (Figure 7A); however, they did significantly decrease SC hydration (Figure 7B). KR slightly increased SC integrity in photoaging skin, and Tes increased it significantly compared to vehicle (Figure 7C).

### 2.7. A Mixture of Extracts of Kochia scopiaria and Rosa multiflora (KR) Did Not Change the Increase in Epidermal Proliferation in Photoaging Skin, Nor Did it Affect Epidermal Differentiation in Photoaging Skin

**Figure 8:**
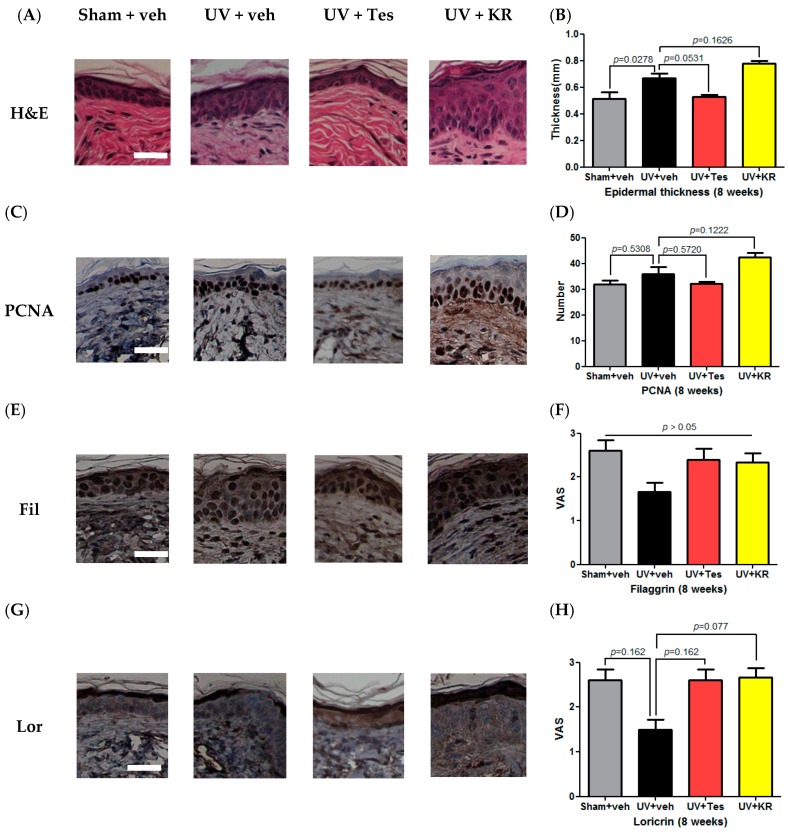
Effect of a mixture of extracts of *Kochia scoparia* and *Rosa multiflora* (KR) on epidermal proliferation and differentiation in photo-aging skin. To study the effects of KR on epidermal proliferation, epidermal thickness was evaluated by hematoxylin and eosin (H & E) staining (**A**); An 8-week course of UV radiation on 18-week-old mice significantly increased epidermal thickness. KR did not change epidermal thickness, whereas Tes showed a tendency to decrease it (**B**); Staining for proliferating cell nuclear antigen (PCNA) was also done to evaluate epidermal proliferation (**C**); UV radiation increased the number of PCNA stained keratinocytes. Neither KR nor Tes changed the number of stained keratinocytes (**D**); Epidermal differentiation represented by filaggrin and loricrin was evaluated after UV radiation accompanied with topical application of KR, vehicle, or tesaglitazar (Tes) for 8 weeks. Photoaging skin did not show any significant decrease in the expression of filaggrin and loricrin in the epidermis. Neither KR nor Tes affected filaggrin (Fil) or loricrin (Lor) expression in photoaging skin (**E**–**H**). The degree of staining was evaluated using a visual analogue scale (VAS) ranging from 0 to 3: 0—no staining, 1—mild staining, 2—moderate staining, 3—strong staining; Scale bars = 100 µm.

To examine the effect of KR on epidermal proliferation in photoaging skin, epidermal thickness was measured with H&E staining and epidermal proliferation with proliferating cell nuclear antigen (PCNA) staining. Eight weeks of UV radiation increased epidermal thickness compared to sham light. KR did not alter the increased epidermal thickness in photoaging skin, whereas Tes decreased it compared to vehicle, although not significantly (Figure 8A,B). In photoaging skin, the number of PCNA-stained keratinocytes increased compared to sham light-treated skin. Neither KR nor Tes altered it relative to vehicle treatment (Figure 8C,D). KR did not affect epidermal proliferation in photoaging skin. We evaluated the expression of epidermal differentiation marker proteins, such as loricrin and filaggrin, using immunohistochemical staining. In photoaging skin, epidermal differentiation did not change. Neither KR nor Tes affected epidermal differentiation compared to vehicle (Figure 8E–H). These findings indicate that KR does not induce remarkable epidermal side effects—such as scale, epidermal thickening, and erythema—which are usually of concern with antiaging agents such as retinoids.

### 2.8. A Mixture of Extracts of Kochia scoparia and Rosa multiflora (KR) Decreased the Expression of Epidermal IL-1α in Photoaging Murine Skin

**Figure 9:**
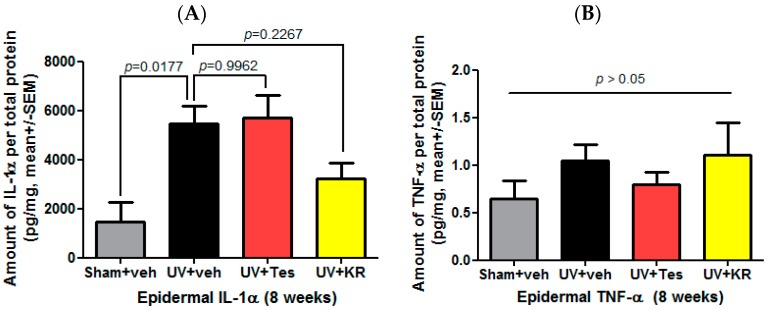
Effect of a mixture of extracts of *Kochia scoparia* and *Rosa multiflora* (KR) on cytokine expression in the epidermis of photoaging murine skin. Epidermal expression of IL-1α and tumor necrosis factor (TNF)-α, represented by the expression of proinflammatory cytokines, was determined by a cytokine assay using a Milliplex kit after topical application of KR, vehicle (veh), or tesaglitazar (Tes) for 8 weeks. In photoaging skin, epidermal IL-1α (**A**) and TNF-α (**B**) increased. KR decreased epidermal IL-1α (**A**), but not epidermal TNF-α (**B**).

To measure cytokine expression in photoaging murine skin, we confirmed the expression of epidermal IL-1α and epidermal tumor necrosis factor (TNF)-α. UV radiation significantly increased the expression of epidermal IL-1α compared to sham light. In photoaging skin, KR decreased epidermal IL-1α expression compared to vehicle, but Tes did not affect it (Figure 9A). Neither KR nor Tes affected the expression of epidermal TNF-α in photoaging skin (Figure 9B).

## 3. Discussion

While searching for natural herbs with PPAR α/γ dual agonistic effects, *Kochia scoparia* and *Rosa multiflora* were selected via in vitro screening tests. Mormodin and ursolic acid are the active constituents of *Kochia scoparia* and *Rosa multiflora*, respectively. We then performed in vivo tests to investigate whether and how a mixture of the extracts of *Kochia scoparia* and *Rosa multiflora* (KR) has an antiaging effect on photoaging skin. In this study, in order to set up a photoaging model modified from previous reports, we used female hairless mice (18 weeks old) exposed to UV light for 8 weeks [20,21]. We elucidate that *Kochia scoparia* (Ks) and *Rosa multiflora* (Rm) have a dual activation of PPAR α/γ transcriptional activity. Therefore, we believe that their mixture (KR), not only inhibits the increase in MMPs and ROS induced by UV light, but also suppresses inflammatory cytokines, as previously reported [17]. KR also increases the number of dermal fibroblasts and dermal thickness through procollagen 1 production induced by TGF-β as shown in this study.

Our study demonstrated that KR increased dermal thickness as well as increased the number of dermal fibroblasts which were decreased by UV radiation. Although KR did not increase dermal collagen densities significantly, it increased the levels of type 1 procollagen protein and mRNA. On the other hand, KR significantly decreased the expression of MMP-13 mRNA. These dermal effects could be construed as an increase in collagen synthesis caused by fibroblast proliferation. In aged skin, there is a decrease in procollagen synthesis and an increase in extracellular matrix degradation as a result of increased matrix metalloproteinase activity [22]. Collagen is the major constituent of the dermis and is made of collagen fibers synthesized by fibroblasts [23]. The expression of procollagen type 1 and 3 in murine skin provides tensile strength and mechanical resistance to the skin [24]. KR promoted the proliferation of dermal fibroblasts and the synthesis of procollagen type 1. Increase in procollagen type 1 production leads to the increase of collagen fibers’ deposition as procollagen as the precursor of collagen. When collagen fibers are newly synthesized, they are of high density in the dermal collagen, increasing dermal thickness and enhancing the appearance of aged skin by reducing wrinkles [25]. Therefore, these results demonstrate that KR increases collagen production by increasing TGF-β, and decreases the expression of collagenase in photoaging murine skin. With respect to dermal effects, KR increases TGF-β expression, which in turn restores the activity of dermal fibroblasts weakened by chronic UV radiation and promotes collagen synthesis, whereas it inhibits MMP-mediated collagen degradation.

In this study, UV radiation did not change basal TEWL, SC hydration, and SC integrity. From these results, we inferred that long-term exposure to a low dose of UV radiation did not seem to have a significant effect on skin barrier function in chronologically young skin [26]. Moreover, neither KR nor tesaglitazar affected basal TEWL, but both significantly decreased SC hydration compared to UV-irradiated vehicle-treated skin. On the contrary, KR slightly increased SC integrity in photoaging skin, whereas Tes increased it significantly compared to UV-irradiated vehicle-treated skin. As a representative marker of the epidermal permeability barrier, SC integrity is directly dependent on the lysis of corneodesmosome (CD), which is involved in the desquamation of corneocytes, a process that is influenced by SC pH. In normal epidermis, the upper SC has a pH of around 4.5–5.0, but the lower SC has a pH of 6.5–7.0, which normally results in a pH gradient in the SC [27]. Serine proteases, specific enzymes in the epidermis, are activated under neutral pH and then contribute to the desquamation of corneocytes and the disruption of the skin barrier [28]. SC hydration is dependent on several factors: natural moisturizing factors (NMFs), lamellar structure of SC intercellular lipids that trap water within the corneocytes, and SC glycerol content. The NMFs are a complex mixture of low molecular weight compounds produced by filaggrin degradation within corneocytes. Although it is known that SC hydration usually changes according to the epidermal permeability barrier state, presented by basal TEWL and SC integrity, an exceptional case has been reported [29]. In this study, both KR and Tes reduced SC hydration without impairing the epidermal permeability barrier. KR did not change the level of epidermal proliferation increased by UV radiation, nor did it affect epidermal differentiation in photoaging skin. These findings imply that KR does not induce notable epidermal side effects—such as scale, epidermal thickening, and erythema—which are usually a concern associated with the use of antiaging agents such as retinoids [30].

Generally, UVR exposure stimulates keratinocytes to secrete abundant proinflammatory cytokines. In the photoaging skin in this study, dermal IL-1β and TGF-β did not change. Epidermal IL-1α and TNF-α increased. KR significantly increased dermal TGF-β, but not dermal IL-1β. KR also decreased epidermal IL-1α, but not epidermal TNF-α. These results show that KR with PPAR α/γ dual agonistic effect stimulated dermal fibroblasts to produce TGF-β and inhibited epidermal keratinocytes from secreting IL-1α.

Because KR differentially affected the epidermis and dermis in photoaging skin, it is considered to be more beneficial for photoaging skin compared to other antiaging agents. For instance, the use of retinoids is the only class of therapy with irrefutable evidence of effectiveness. Topical retinoids reduce MMP expression and stimulate collagen synthesis in aged sun-protected skin, as it does in photoaged skin [31]. However, retinoids also induce epidermal hyperplasia, leading to uncomfortable side effects such as erythema, peeling, and stinging [31]. Although these symptoms of skin irritation decline with continued use, retinoid-induced scaling caused by epidermal hyperplasia is still the major deterrent for its topical use [32].

In conclusion, KR, with its PPAR α/γ dual agonistic effect, prevented the dermal aging process by increasing the number of dermal fibroblasts and dermal thickness through procollagen 1 production induced by TGF-β and by decreasing collagen degradation through inhibition of MMP-13. In addition, it did not induce epidermal side effects associated with barrier impairment and epidermal hyperplasia.

## 4. Materials and Methods

### 4.1. Plant Materials and Their Extraction

Both *Kochia scoparia* (Ks) and *Rosa multiflora* (Rm) were purchased from a local market (Kyungdong Herb-Market, Seoul, Korea) and authenticated by K.H. Kim of Sungkyunkwan University, Suwon, Korea. Voucher specimens (KIST-821-15 and KIST-818-15) have been deposited in the herbarium of KIST Gangneung Institute, Gangneung, Korea. The dried fruits (1 kg) of Ks were extracted with methanol under reflux to yield 88.4 g of residue. The methanol extracts of Ks were suspended in water and then partitioned in turn by methylene chloride, ethyl acetate, and *N*-butanol. After evaporation and dehydration of the solvent, methylene chloride (12.5 g), ethyl acetate (28.4 g), *N*-butanol (13.5 g), and water (32.1 g) fractions were obtained. The dried seeds (1 kg) of Rm were extracted with methanol under reflux to yield 62.5 g of residue. The methanol extracts of Rm were suspended in water and then partitioned in turn by methylene chloride, ethyl acetate, and *N*-butanol. After evaporation and dehydration of the solvent, methylene chloride (11.4 g), ethyl acetate (19.2 g), *N*-butanol (12.7 g), and water (18.0 g) fractions were obtained.

### 4.2. Cell-Based Transactivation Assay

CV-1 cells, derived from Cercopithecus monkey kidneys, were obtained from American Type Culture Collection (Manassas, VA, USA), cultured in 24-well plates for 24 h before transfection. Then, we changed the medium to 10% charcoal dextran-treated fetal bovine serum (FBS)/Dulbecco’s modified Eagle’s medium (DMEM). Four hours after the medium changed, a DNA mixture was transfected into the cells using the TransFast™ transfection reagent (Promega, Madison, WI, USA). The DNA mixture included a 3X multimerized peroxisome proliferator response element (PPRE)–luciferase reporter plasmid (0.3 µg), pcDNA3-hPPAR (30 ng), and an internal control plasmid, pRL-SV-40 (5 ng). After 24 h of transfection, the cells were treated with 10 µM WY14643, 10 µM troglitazone or the indicated fractions of Ks or Rm and incubated for an additional 24 h. Having followed the manufacturer’s instructions (Promega), the cell lysates’ luciferase activities were measured with the use of the Dual-Luciferase^®^ Reporter Assay System. To determine the transfection efficiency, the relative luciferase activity was normalized to the corresponding Renilla luciferase activity.

### 4.3. UVB Irradiation and Enzyme-Linked Immunosorbent Assay (ELISA)

Normal human fibroblasts (NHFs) were obtained from Lonza (Basel, Switzerland) and were cultured in Dulbecco’s modified Eagle’s medium (DMEM; Invitrogen, Carlsbad, CA, USA) supplemented with 10% fetal bovine serum (FBS; HyClone Laboratories Inc., Logan, UT, USA), penicillin (100 U/mL), and streptomycin (100 µg/mL) (Invitrogen) at 37 °C and 5% CO_2_. Cells were cultured in a 48-well plate (7.0 × 10^4^ cells/well). After 24 h of serum starvation, the cells were washed with phosphate-buffered saline (PBS), treated with the methanol chloride (MC) fraction of Ks or Rm for 1 h, and exposed to UVB irradiation (20 mJ/cm^2^) through a thin layer of PBS. After UVB irradiation, cells were incubated with the MC fraction of Ks (10 µg/mL) or Rm (10 µg/mL) for another 48 h. MMP-1 secretion was quantified from supernatants using a human MMP-1 ELISA Kit (Merck & Co., Inc., Whitehouse Station, NJ, USA). The relative MMP-1 amount was normalized to the corresponding viability of cells determined by the MTT assay (Ez-cytox, Dail Lab Service. Co., Seoul, Korea).

### 4.4. Animal Model for Photoaging Skin

All animal procedures (YWC-131205-1) were approved on 12 December 2013 by the Yonsei University Wonju Campus Institutional Animal Care and Use Committee (IACUC). Twenty-four female hairless mice (SKH-1) were purchased from Orient Bio (Seongnam, Korea) and were reared up to 18 weeks old in a standard environment with the temperature maintained at 22 ± 2 °C, relative humidity at 40% ± 5%, and a 12 h/12 h light and dark cycle. The mice were irradiated with a 1/2 minimal erythema dose (MED) (UVA: 14 J/cm^2^, UVB: 40 mJ/cm^2^) three times a week for 8 weeks. UVB and UVA radiation were delivered as previous reported [20]. Philips TL20W/12RS lamps (Philips, Utrecht, The Netherlands) and Philips CLEO performance 40 W lamps (Philips) have the wavelength of between 290 to 315 nm (UVB) and 315 to 380 nm (UVA), respectively.

### 4.5. Topical Treatment with a Mixture of the Extracts of Kochia scoparia and Rosa multiflora (KR)

The mice were randomly divided into four groups (each *n* = 6). The first group (sham + veh) was treated with 50 µL of vehicle (propylene glycol:EtOH = 7:3, *v*/*v*) twice a day during an 8-week sham light radiation period. The second group (UV + veh) was treated with 50 µL of vehicle, the third group (UV + Tes) was treated with 50 µL of tesaglitazar, a known PPAR α/γ dual agonist, and the fourth group (UV + KR) was treated with 50 μL of a mixture of the 1% extracts of *Kochia scoparia* and *Rosa multiflora* (KR) twice a day during the 8-week UV radiation period.

### 4.6. Evaluation of Skin Barrier Function

After 8 weeks of UV radiation and topical application, basal transepidermal water loss (TEWL) was measured using the Tewameter TM210 device (Courage and Khazaka, Cologne, Germany) and stratum corneum (SC) hydration was assessed as capacitance with the Corneometer CM820 device (Courage and Khazaka) on the dorsal surface of the mice, as described previously [33]. Measurements were performed under anesthesia with intraperitoneal injections of 4% chloral hydrate. SC integrity was determined by measuring TEWL after four sequential strippings with D-Squame^®^ disks (CuDerm, Dallas, TX, USA) [34].

### 4.7. Tissue Preparation and Microscopic Measurement

Skin specimens obtained 24 h after the last treatment were fixed in 10% formalin and embedded in paraffin. Subsequently, tissue microarrays (TMA) (AccuMax™ Array; ISU ABXIS, Seongnam, Korea) were performed. Six micrometer thick TMA sections were stained with hematoxylin and eosin (H & E) and pictures of each section were taken under 200× magnification. Dermal thickness, defined as the distance between subcutaneous fat and the dermo-epidermal junction, and epidermal thickness, defined as a distance between the basement membrane and the SC–stratum granulosum (SC–SG) junction, were measured randomly at five sites on each picture. Fibroblasts were counted in two areas of each picture (perfect squares of the same area; Photoshop software, Adobe Systems, Mountain View, CA, USA). The collagen density, which was visualized with Masson-trichrome staining, was analyzed with a histogram created with Photoshop software.

### 4.8. Immunohistochemistry for Epidermal Differentiation and Proliferation Markers

Paraffin-embedded TMA sections were used for immunohistochemistry. Immunohistochemical stains were prepared as described in previous reports [20,21]. After deparaffinization, the sections were rehydrated with a sequential treatment of 100%, 90%, and 70% ethanol solutions. Then, they were incubated with a peroxidase-blocking reagent for 30 min to avoid endogenous peroxidase activity, and with a serum-free protein block for 15 min at room temperature (RT) to block nonspecific antibody binding. They were applied with primary antibodies of loricrin (Abcam, Boston, MA, USA) and filaggrin (Abcam) for 30 min at 37 °C. Loricrin and filaggrin were detected using diaminobenzidine (DAB) as a substrate. The degree of staining was measured by scoring system from 0 to 3 (0: no staining; 1: mild; 2: moderate; 3: strong) and reported as the mean ± SEM. Keratinocyte proliferation was stained as PCNA-positive cells with anti-PCNA antibody (Abcam) and counted in the basal and supra-basal cell layers from each section.

### 4.9. Quantification of Procollagen Type 1 and Matrix Metalloproteinase-13 (MMP-13) Expression by Enzyme-Linked Immunosorbent Assay (ELISA) Analysis

Procollagen type 1 and MMP-13 were measured by ELISA and quantified in nanograms of protein. All protein samples were extracted from murine skin using the Qproteome Mammalian Protein Prep kit (Qiagen, Hilden, Germany), according to the manufacturer’s manual. The concentration of protein was determined by the Bradford assay [34]. The expression level of procollagen type 1 was determined with a mouse procollagen type 1 N-terminal propeptide (PINP) ELISA kit (MyBioSource, San Diego, CA, USA). The expression level of MMP-13 was determined with a mouse matrix metalloproteinase 13 (MMP-13) ELISA kit (MyBioSource). Samples and standards were incubated together with horseradish peroxidase (HRP)-conjugated reagent in pro-coated plates for 1 h. After the incubation, the wells were decanted and washed five times. The wells were then incubated with a substrate for the HRP enzyme. Finally, a stop solution was added to stop the reaction. The intensity of color was measured at 450 nm in a microplate spectrophotometer (BioTek Instruments, Inc., Winooski, VT, USA). The concentration of total protein was determined by comparing the optical density (OD) of the samples to the standard curve. The measurement was carried out in triplicate.

### 4.10. Total RNA Preparation and cDNA Synthesis Using Murine Skin

Methods described in our previous report [35] were also used in performing total RNA preparation and cDNA synthesis. Monophasic solution of phenol and guanidine isothiocyanate (TRIzol Reagent, Invitrogen, Carlsbad, CA, USA) were used for the extraction of total RNA. RNA concentration was determined by a UV-visible spectrophotometer (Amersham Biosciences, Piscataway, NJ, USA) at 260 nm. Aliquots (1.0 µg) of RNA from each sample were reverse-transcribed using Moloney murine leukemia virus reverse transcriptase (MML-V RTase, Promega). 1× RT-buffer, 2 mM deoxynucleotide triphosphates (dNTPs, Promega), 0.2 pM oligo dT primer (16-mer) (Bioneer Inc., Daejeon, Korea), and MML-V RTase (2.5 units/µL) in 20 µL reaction volumes were added in performing reverse transcription. Then, the samples were incubated at 42 °C for 60 min and then stored at −20 °C.

### 4.11. Quantitative PCR Analysis of Gene Expression

Expression of specific mRNAs was quantified using a Rotor-Gene™ 3000 (Corbett Life Science, Brisbane, Australia), as described in our previous report [35]. Briefly, we set up 10-μL PCR reactions including 0.5 pM/µL each of probes and primers, Quantitect SYBR green PCR kit master mix (Qiagen) in a 2× solution, 8 mM manganese chloride, 200 µM dNTPs, and 1.25 units Hotstart Taq polymerase. About 60 ng of cDNA per reaction was used. Based on GeneBank sequences using Primer 3 software (http://frodo.wi.mit.edu/cgi-bin/primer3/primer3.cgi/, Massachusetts Institute of Technology, Cambridge, MA, USA), we designed the primers for procollagen type 1 and MMP-13. GAPDH (glyceraldehyde-3-phosphate dehydrogenase) was used as a housekeeping gene in all reactions. According to the manufacturer’s guidelines, data were obtained as *C*_t_ values (the cycle number that logarithmic PCR plots cross a calculated threshold line) and used to determine the ∆*C*_t_ values (*C*_t_ of target gene − *C*_t_ of GAPDH) as raw data for gene expression. Fold changes in gene expression were determined by subtracting ∆*C*_t_ values for the samples from their respective control samples in all groups. Then, to calculate the fold change in gene expression as 2^−∆∆*C*t^, the resulting ∆∆*C*_t_ values were used. We performed all reactions in triplicate, and expressed the results as the mean of values from three separate experiments. We amplified the samples under the conditions: 95 °C for 15 min, 45 cycles at 95 °C for 15 s, and 60 °C for 1 min.

### 4.12. Cytokine Assays

Cytokine assays were performed using the Milliplex^®^ Map Mouse Cytokine/Chemokine Magnetic Bead Panel kit (Millipore, Billerica, MA, USA) and Milliplex™ Map TGFβ1 Single Plex Kit on a Luminex^®^ 200 multiplex system (Invitrogen, Carlsbad, CA, USA). According to the manufacturer’s instructions, we prepared all reagent dilutions (beads, cytokine standards, cytokine controls, biotinylated detection antibody, etc.). According to the vendor-provided instructions, we performed cytokine assays. We incubated the cytokine standards, controls, samples, and microspheres on a rotator in 96-well filter bottom microtiter plates (Millipore) at RT for 2 h to allow for subsequent washing. After an additional 30 min incubation on the rotator and washing, we added 100 µL of streptavidin (10 µg/mL)-conjugated R-phycoerythrin (Invitrogen) to each well. After a 30 min incubation and a final wash, we resuspended the microspheres in 200 µL of washing buffer, and placed the 96-well microplate in a Luminex 100 instrument. We determined the amount of cytokine bound to the microsphere as the median fluorescence intensity (MFI) of the reporter molecule, phycoerythrin. Then, the MFI of unknown samples was converted into picograms per milliliter based on the known cytokine concentrations of the standard curve using a five-parameter regression formula.

### 4.13. Statistics

Normality tests were used to determine if all data sets were well-modeled by a normal distribution. Statistical analyses were performed using ANOVA and Scheffe test for measurements and Kruskal Wallis test for immunohistochemical stains. All data were calculated as mean ± SEM. Differences in values were considered significant if *p* < 0.05.

## Abbreviations

PPAR	peroxisome proliferator activated receptor
KR	a mixture of extracts of *Kochia scoparia* and *Rosa multiflora*
UV	ultraviolet
TEWL	transepidermal water loss
SC	stratum corneum
FDA	Food and Drug Administration
AD	atopic dermatitis
TIMP	tissue inhibitor of metalloprotease
